# Association Between Triglyceride/High-Density Lipoprotein Ratio and Incidence Risk of Heart Failure: A Population-Based Cohort Study

**DOI:** 10.3390/jcm14030950

**Published:** 2025-02-01

**Authors:** Yoon-Kyung Chang, Ju-Young Park, Tae-Jin Song

**Affiliations:** 1 Department of Neurology, Mokdong Hospital, Ewha Womans University College of Medicine, Seoul 07804, Republic of Korea; ykchang@ewha.ac.kr; 2 Department of Applied Statistics, Yonsei University, Seoul 03722, Republic of Korea; 3 Department of Statistics and Data Science, Yonsei University, Seoul 03722, Republic of Korea; ka2pzy@naver.com; 4 Department of Neurology, Seoul Hospital, Ewha Womans University College of Medicine, Seoul 07804, Republic of Korea

**Keywords:** insulin resistance, triglyceride/high-density lipoprotein ratio, heart failure, diabetes mellitus

## Abstract

**Background/Objectives**: The triglyceride/high-density lipoprotein (TG/HDL) ratio serves as a simple marker for insulin resistance. We investigated whether the TG/HDL ratio would be associated with the incidence risk of heart failure (HF). **Methods**: The study utilized data from the National Health Insurance Service-Health Screening Cohort database of South Korea from 2002 to 2019. The TG/HDL ratio was utilized as a time-dependent covariate or average value of at least three times throughout the follow-up period. The outcome of interest was incident heart failure (HF) corresponding with the International Classification of Disease, Tenth Revision code of I50. **Results**: A total of 293,968 individuals were included in this study. During the median 9.6 years (interquartile range 9.2–10.13), 27,852 individuals (9.47%) had a cumulative incidence of HF. Considering the multivariable time-dependent Cox proportional hazard model with the repeated measures of the TG/HDL ratio, per unit increase in the TG/HDL ratio significantly increased the risk of HF in the entire cohort (hazard ratio (HR): 1.007, 95% confidence interval (CI): 1.002–1.011), diabetes mellitus (DM) cohort (HR: 1.006. 95% CI: 1.002–1.010), and non-DM cohort (HR: 1.008, 95% CI: 1.003–1.013). Regarding average TG/HDL ratio quartiles, compared to the lowest quartiles (Q1), the highest quartiles (Q4) were positively associated with the incidence risk of HF accompanied by a significant *p* for trend (HR: 1.114, 95% CI: 1.075–1.155) in fully adjusted multivariable analysis. **Conclusions**: Our study demonstrated that the repeatedly measured TG/HDL ratio was associated with the incidence risk of HF regardless of the presence of DM history in the general population.

## 1. Introduction

Heart failure (HF) is a clinical condition characterized by the heart’s reduced ability to pump blood effectively, leading to challenges in either blood ejection or ventricular filling [1]. This condition is a common cardiovascular disorder globally and presents a significant health burden due to its increasing prevalence [2]. Despite advances in treatment and prevention, HF still results in substantial morbidity and mortality [2]. Recognizing and understanding risk factors for HF is crucial for managing and potentially mitigating its impact. Known risk factors include hypertension, diabetes mellitus (DM), coronary artery disease, aortic atheroma, poor oral hygiene, and smoking. These factors highlight the importance of addressing modifiable lifestyle and health conditions to reduce the risk of HF [3,4,5,6]. However, the understanding of HF is continually evolving, and there is a need for more research to uncover additional modifiable risk factors.

Insulin resistance is a metabolic disorder commonly linked with type 2 DM [7]. This condition occurs when the body’s cells become less responsive to insulin, a hormone essential for regulating blood sugar levels [8]. The implications of insulin resistance extend beyond DM, affecting many health issues. It is closely associated with various diseases or poor prognoses, such as hypertension, dyslipidemia, liver diseases, cardiovascular diseases, neurodegenerative diseases, certain types of cancer, obesity, and inflammatory and infectious diseases [8,9,10,11].

The triglyceride/high-density lipoprotein (TG/HDL) ratio serves as a simple and practical surrogate marker for insulin resistance [12,13]. This index has gained recognition for its ease of use and cost-effectiveness, especially in settings where more direct and complex measurements of insulin resistance are not readily available. It provides a valuable tool for assessing metabolic health in various clinical settings and identifying individuals at risk of developing complications associated with insulin resistance [14,15,16].

To date, there have been few studies on whether the increase in insulin resistance may increase the incidence risk of HF. Additionally, although insulin resistance can be changed, studies using repeatedly measured TG/HDL ratios in the general population have been limited. We hypothesize that an increased TG/HDL ratio would be associated with the development of HF. This study aimed to assess the association between the TG/HDL ratio and the incidence of heart failure. We investigated whether the TG/HDL ratio is independently associated with the risk of heart failure in a longitudinal setting in the general population.

## 2. Materials and Methods

This study sourced its data from the National Health Insurance Service-Health Screening Cohort (NHIS-HEALS) database, a subset of the Korean National Health Insurance Service (NHIS). The NHIS, a government program, provides health insurance to nearly 97% of the Korean population. The Medical Aid program, an affiliate of the NHIS, attends to the 3% of the population not covered by the NHIS. Our study was conducted based on the NHIS-HEALS cohort database of South Korea (2002–2019) [17]. The NHIS provides a nationwide free health screening program every two years for all South Korean adults aged 40 and over.

The NHIS-HEALS encompassed measurements of blood pressure, the body mass index, blood biochemistry, a self-administered questionnaire on medical history, and lifestyle including smoking habits, alcohol consumption, and physical activity. Additionally, health claim data covering all hospital visits, diagnoses, surgeries, medical procedures, and prescriptions of participants from 2002 to 2019 were included. Diagnoses at each hospital visit were recorded based on the International Classification of Disease, Tenth Revision (ICD-10). Demographic information such as sex, age, and household income were also included, and data regarding participants’ health claims, insurance coverage maintenance, and death were available up to 31 December 2019.

**Figure 1:**
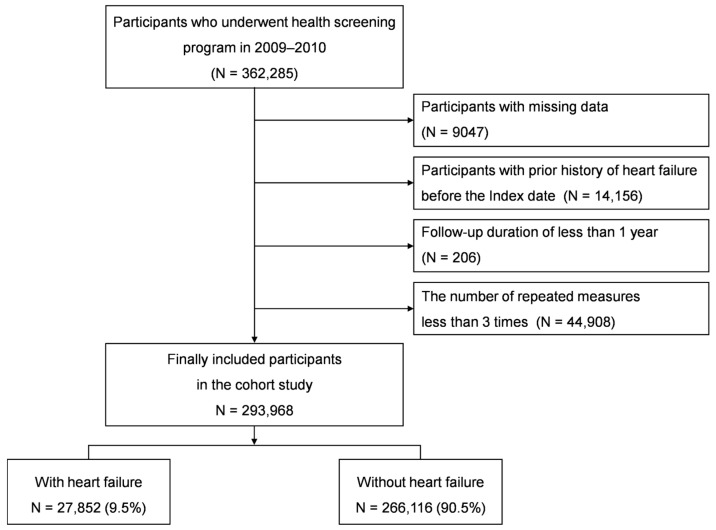
Flow chart of inclusion and exclusion criteria.

From the NHIS-HEALS database, we included 362,285 participants aged 40 and over who participated in the national health screening program during the baseline years of 2009–2010. Among 362,285 participants, those with missing data on demographic information, lifestyle, and laboratory findings were excluded (*n* = 9047). The washout period extended from 2002 up to the index date, during which patients with a history of HF occurrence were excluded (n = 14,156). Participants with a follow-up duration of less than 1 year (n = 206) for excluding reverse causality or association and participants with less than 3 repeated measurements (n = 44,908) were excluded. After applying these inclusion and exclusion criteria, the final cohort for analysis comprised 293,968 individuals (Figure 1).

Based on health claim data from the NHIS-HEALS, participants’ demographic information (age, sex, body mass index (BMI), waist circumference, household income) and lifestyle (smoking status, alcohol consumption, regular physical activity) were collected through self-reported questionnaires. BMI was calculated as weight (kg)/height (m)^2^. Household income was categorized using quantiles of the individual’s health insurance premiums, with those in the 9th decile and above considered high income. Lifestyles were detailed as follows: Smoking status was categorized into never, former, and current smokers. The frequency of alcohol consumption was defined by the number of times alcohol was consumed per week: none, 1–2 times, 3–4 times, and ≥5 times. The frequency of regular physical activity was divided based on the number of days engaged in exercise per week: none, 1–4 days, and ≥5 days. Biochemical measurements included liver enzyme, lipid panel, and fasting glucose collected from the health screening laboratory results. Hypertension, DM, dyslipidemia, renal disease, and liver disease were considered as comorbidities, and the Charlson comorbidity index (CCI) was taken into account for the burden of covariates. Detailed definitions for these can be found in the Supplementary Methods [18,19,20,21,22,23,24].

In this study, the TG/HDL ratio was considered as a time-dependent covariate throughout the follow-up period. To enhance reliability and reduce bias, the analysis was conducted on individuals who had their TG/HDL ratio measured at least three times. The average of all repeated measurements was used for the analysis.

For measuring outcome, the index date was defined as the date of the most recent health examination. The outcome was based on individuals who had filed one or more insurance claims for ICD-10 code (I50) for HF [6,25]. In our study, individuals who had previously been diagnosed with HF or had claims for HF treatment before the index date were not considered as the incidence of HF. This means that the definition of incidence of HF in our study referred to those who were hospitalized and made their first claim for HF treatment after the index date. Follow-up was carried out until 31 December 2019, death, or the first occurrence of HF.

Comparisons between groups based on quartiles of the TG/HDL ratio were made using one-way ANOVA (Analysis of Variance), the Bonferroni Correction for continuous variables, and the Chi-squared test (or Fisher’s exact test) for categorical variables. The survival curves for the time-to-event outcomes were plotted using Kaplan–Meier curves, and the log-rank test was used to compare the survival curves across the quartile of TG/HDL ratio groups.

To evaluate the incidence risk of HF concerning the repeatedly measured TG/HDL ratio during the follow-up period, the time-dependent Cox proportional hazard model was applied. Furthermore, participants were divided into four groups based on the quartiles (Q) (Q1, Q2, Q3, and Q4) of the average TG/HDL ratio during the follow-up period. To ascertain the risk of HF according to quartile groups, the conventional Cox proportional hazard model was utilized. The proportionality of the hazard assumption was evaluated using the Grambsch and Therneau test of Schoenfeld residuals, which yielded satisfactory results.

The results of time-dependent Cox regression and conventional Cox regression analysis were demonstrated as hazard ratios (HR) and 95% confidence interval (CI) for an unadjusted model, model 1, and model 2, depending on the adjustment of covariates. Model 1 was adjusted for age and sex, while model 2 was additionally adjusted for model 1 + BMI, household income, smoking status, alcohol consumption, regular physical activity, hypertension, DM, renal disease, liver disease, and CCI. Blood biomarkers were not additionally adjusted in multivariable model 2 due to multi-collinearity such as liver enzyme and liver disease. Considering covariates, in cases where participants underwent multiple health check-ups from 2009 to 2019, the data from their latest examination were utilized for statistical analysis. For the sensitivity analysis, due to insulin resistance being closely associated with DM, we performed further analysis according to the presence of DM. Subgroup analyses regarding the association of the TG/HDL ratio with HF were performed according to demographics, lifestyle, and covariates, suggesting a *p*-value for interaction. All statistical analyses were conducted using SAS version 9.4 (SAS Inc., Cary, NC, USA) and R software, version 4.2.1 (R Foundation for Statistical Computing, Vienna, Austria), with statistical significance defined as a two-sided *p*-value < 0.05.

## 3. Results

### 3.1. Baseline Characteristics of Participants

The number of measurements repeated during the follow-up period is described in Supplementary Table S1, and the characteristics of variables for each year are described in Supplementary Table S2.

Table 1 presents the baseline characteristics of the entire cohort divided into four groups based on the quartiles of the average TG/HDL ratio (Q1 (<1.585), Q2 (1.585–2.305), Q3 (2.305–3.403), and Q4 (≥3.403)). The Q3 group had the highest proportion of individuals aged over 65 years (21.2%, *p* < 0.001). Women (67.6%, *p* < 0.001) and individuals with a BMI ≥ 25 kg/m^2^ (46.7%, *p* < 0.001) were more prevalent in the Q4 group. Additionally, the Q4 group reported a higher income level compared to other groups (36.7%, *p* = 0.002). Notably, the Q4 group also had a greater prevalence of current smokers (26.0%, *p* < 0.001) and high alcohol consumption (≥5 days/week, 5.7%, *p* < 0.001), while engaging less frequently in exercise (no sessions, 24.9%, *p* < 0.001). Regarding comorbidities, the Q4 group exhibited significantly higher frequencies of hypertension (35.3%, *p* < 0.001), DM (17.2%, *p* < 0.001), dyslipidemia (18.5%, *p* < 0.001), renal disease (14.2%, *p* < 0.001), liver disease (18.4%, *p* < 0.001), and a CCI score of 2 or more (6.8%, *p* < 0.001).

### 3.2. Relationship of TG/HDL Ratio with Incidence Risk for HF

**Figure 2:**
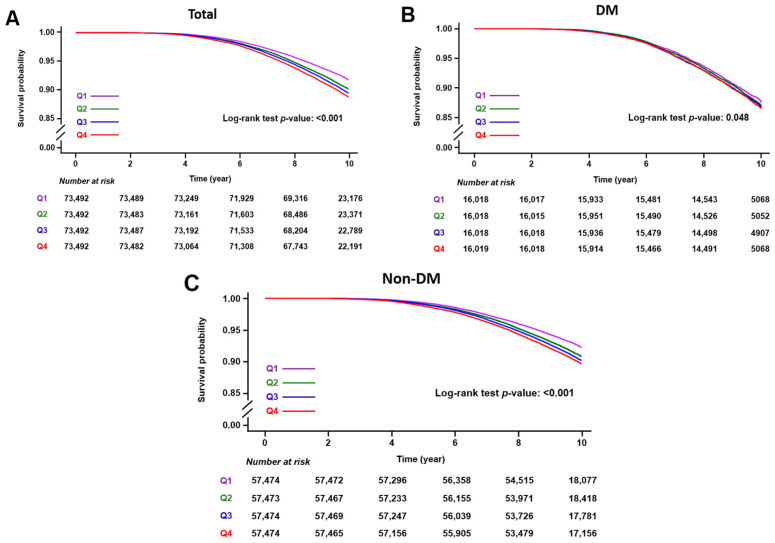
Kaplan–Meier survival curves of heart failure outcome according to TG/HDL ratio quartiles. (**A**) Total cohort. (**B**) Diabetic mellitus cohort. (**C**) Non-diabetic mellitus cohort.

During the median 9.6 years (interquartile range 9.2–10.13), 27,852 individuals (9.47%) had a cumulative incidence of HF. Survival curves depicting the incidence of HF across quartiles of the average TG/HDL ratio are presented in Figure 2. Higher TG/HDL ratio quartiles (Q1 to Q4) were associated with an increased risk of HF (log-rank test in entire cohort: *p* < 0.001, DM cohort: *p* = 0.048, and non-DM cohort: *p* < 0.001).

Considering the multivariable time-dependent Cox proportional hazard model with the repeated measures of the TG/HDL ratio, per unit increase in the TG/HDL ratio significantly increased the risk of HF in the entire cohort (HR: 1.007, 95% CI: 1.002–1.011), DM cohort (HR: 1.006. 95% CI: 1.002–1.010), and non-DM cohort (HR: 1.008, 95% CI: 1.003–1.013) in fully adjusted multivariable models (Table 2 and Supplementary Table S3).

Results of the multivariable Cox proportional model for average TG/HDL ratio quartiles during follow-up are detailed in Table 3 and Supplementary Table S4. Comparing the lowest quartiles (Q1), the highest quartiles (Q4) were positively associated with the incidence risk of HF accompanied by a significant *p* for trend (HR: 1.114, 95% CI: 1.075–1.155 in the entire cohort; HR: 1.102, 95% CI: 1.043–1.162 in DM cohort; HR: 1.134, 95% CI: 1.087–1.182 in non-DM cohort) in fully adjusted multivariable analysis.

### 3.3. Subgroup Analysis for Association of TG/HDL Ratio with Incidence Risk of HF

**Figure 3:**
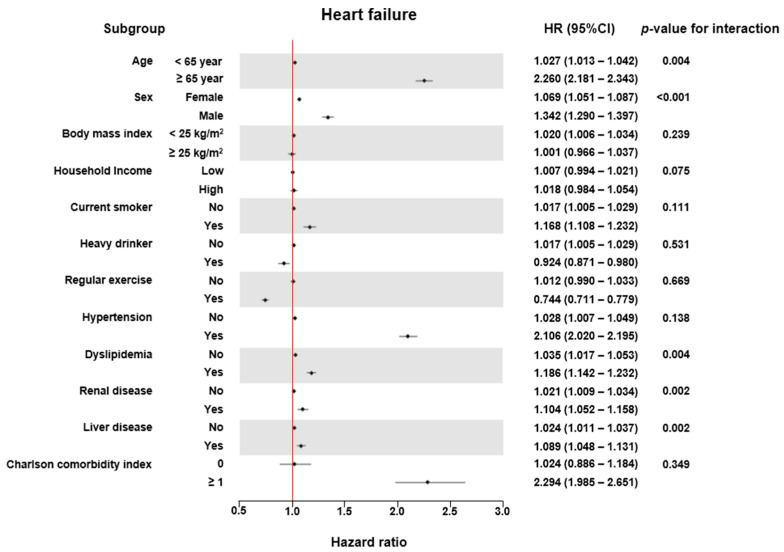
Forest plots of heart failure risk according to the TG/HDL ratio in the various clinical sub-groups.

The association of the TG/HDL ratio with the incidence risk of HF was more significantly noted in the older age group (≥65 years) compared to that in the younger group (<65 years) (*p* for interaction = 0.004), in men compared to women (*p* for interaction < 0.001), in the dyslipidemia group compared to the group without dyslipidemia (*p* for interaction = 0.004), in the renal disease group compared to the group without renal disease (*p* for interaction = 0.002), and in the liver disease group compared to the group without liver disease (*p* for interaction = 0.002) (Figure 3).

## 4. Discussion

The key findings of our study were that the TG/HDL ratio was associated with the incidence risk of HF regardless of the presence of DM history in the general population even in time-dependent analysis and applying repeatedly measured average value of the TG/HDL ratio.

The TG/HDL ratio is linked to several health conditions for presence, progression, and adverse events. For example, an increased TG/HDL ratio has been correlated with a heightened incidence of metabolic syndrome, cerebrovascular disease, coronary artery disease, and peripheral arterial disease [26]. Notably, in patients infected with coronavirus disease 2019, elevated TG/HDL ratio levels have been associated with more severe illness and increased mortality rates [27]. Moreover, previous studies showed a significant association of the TG/HDL ratio with long-term all-cause mortality and the development of HF in patients with coronary artery disease [28,29]. In the cross-sectional setting with the National Health and Nutrition Examination Survey, the TG/HDL ratio was closely associated with the prevalence of HF [30]. Accordingly, our study is meaningful in that it presents additional information regarding the association between the repeatedly measured TG/HDL ratio and incidence risk of HF in the general population with a large sample size and longitudinal setting.

In our subgroup analysis, we observed a heightened association between the TG/HDL ratio and HF in specific demographic and clinical categories, including older individuals, men, and those with dyslipidemia, renal disease, and liver disease. In these subgroups, the relationship between the TG/HDL ratio and HF risk appeared to be particularly pronounced. Consequently, it is crucial to exercise caution when interpreting these findings, as they suggest a potentially heightened risk of HF associated with elevated TG/HDL ratio levels in these demographic and clinical cohorts.

The association between the TG/HDL ratio and HF can be attributed to several underlying mechanisms. The TG/HDL ratio, derived from fasting plasma glucose and TG levels, emerges as a practical and reliable clinical proxy for assessing metabolic syndrome and insulin resistance [31]. Consequently, the observed association between the TG/HDL ratio and HF likely revolves around the mechanism of insulin resistance. Insulin resistance and metabolic dysfunction are widely acknowledged to elevate HF risk [32,33,34,35]. Impaired insulin signaling in the coronary arteries and cardiomyocytes in an insulin-resistant state may influence the occurrence of HF [36]. Moreover, systemic inflammatory markers, such as tumor necrosis factor-α and interleukin-1β, which trigger c-Jun N-terminal kinases and IκB kinase β/Nuclear Factor Kappa B pathways, are linked to insulin resistance, potentially fostering atherosclerosis and vascular remodeling [37]. Additionally, insulin resistance can disrupt Phosphoinositide 3-kinase-dependent signaling, upsetting the balance between nitric oxide production and endothelin-1, ultimately leading to endothelial dysfunction [38]. On the other hand, higher levels of TGs, one of the components of the TG/HDL ratio, are associated with adverse cardiovascular outcomes, including HF [39,40]. Increased TG levels may contribute to the development of atherosclerosis and coronary artery disease, which are significant risk factors for HF [41,42,43].

The findings of our study highlight the potential utility of the TG/HDL ratio as a biomarker in advancing personalized medicine. Integration of the TG/HDL ratio into clinical assessments could facilitate precise risk stratification for HF development. For individuals with elevated TG/HDL ratios, tailored interventions such as lifestyle modifications, pharmacological treatments, and targeted monitoring strategies could be implemented to mitigate HF risk. This personalized approach may enhance therapeutic efficacy while minimizing unnecessary interventions for low-risk individuals. Furthermore, regular monitoring of the TG/HDL ratio could provide valuable insights for dynamic adjustments in preventive and therapeutic strategies, aligning with the individualized health trajectories of patients.

The limitation of this study is that it is difficult to apply our results to other races because it was conducted only on the Korean population. Although the TG/HDL ratio was repeatedly checked several times to increase reliability, it is nevertheless difficult to present a causal relationship because it is a retrospective study. Furthermore, as the study utilized health screening data from the general population, it lacked essential biomarkers commonly associated with HF, such as B-type natriuretic peptide and cardiac enzymes, thereby restricting the ability to adjust for these crucial variables in the analysis. Lastly, evidence from cardiac imaging, such as echocardiography or thallium scans, was not available in this study cohort.

In conclusion, our study demonstrated that the repeatedly measured TG/HDL ratio was associated with the incidence risk of HF regardless of the presence of DM history in the general population. The TG/HDL ratio should be considered as an association factor in the risk of future HF incidence.

## Figures and Tables

**Table 1:** Baseline characteristics of study participants.

Variables	Total	TG/HDL Ratio Quartile				
		Q1 (<1.585)	Q2 (1.585–2.305)	Q3 (2.305–3.403)	Q4 (≥3.403)	*p*-Value
Number	293,968	73,492	73,492	73,492	73,492	
Age (N (%))						<0.001
<65 years	237,495 (80.8)	61,382 (83.5)	58,410 (79.5)	57,928 (78.8)	59,775 (81.3)	
≥65 years	56,473 (19.2)	12,110 (16.5)	15,082 (20.5)	15,564 (21.2)	13,717 (18.7)	
Sex (N (%))						<0.001
Male	134,156 (45.6)	41,877 (57.0)	36,633 (49.8)	31,843 (43.3)	23,803 (32.4)	
Female	159,812 (54.4)	31,615 (43.0)	36,859 (50.2)	41,649 (56.7)	49,689 (67.6)	
Body mass index (N (%))						<0.001
<25 kg/m^2^	193,225 (65.7)	58,935 (80.2)	50,202 (68.3)	44,921 (61.1)	39,167 (53.3)	
≥25 kg/m^2^	100,743 (34.3)	14,557 (19.8)	23,290 (31.7)	28,571 (38.9)	34,325 (46.7)	
Waist circumference (N (%))						<0.001
Male < 90 cm, female < 85 cm	238,289 (81.1)	66,690 (90.7)	61,197 (83.3)	57,215 (77.9)	53,187 (72.4)	
Male ≥ 90 cm, female ≥ 85 cm	55,679 (18.9)	6802 (9.3)	12,295 (16.7)	16,277 (22.1)	20,305 (27.6)	
Household income (N (%))						0.002
Low	187,033 (63.6)	46,658 (63.5)	47,178 (64.2)	46,666 (63.5)	46,531 (63.3)	
High	106,935 (36.4)	26,834 (36.5)	26,314 (35.8)	26,826 (36.5)	26,961 (36.7)	
Smoking status (N (%))						<0.001
Never	188,248 (64.0)	54,785 (74.5)	49,891 (67.9)	45,521 (61.9)	38,051 (51.8)	
Former	55,945 (19.1)	11,677 (15.9)	13,287 (18.1)	14,675 (20.0)	16,306 (22.2)	
Current	49,775 (16.9)	7030 (9.6)	10,314 (14.0)	13,296 (18.1)	19,135 (26.0)	
Alcohol consumption (N (%))						<0.001
None	173,864 (59.1)	46,252 (62.9)	45,551 (62.0)	43,423 (59.1)	38,638 (52.6)	
1–2 times/week	79,201 (26.9)	18,572 (25.3)	18,582 (25.3)	1,9941 (27.1)	22,106 (30.1)	
3–4 times/week	26,989 (9.3)	5577 (7.6)	6109 (8.3)	6764 (9.2)	8539 (11.6)	
≥5 times/week	13,914 (4.7)	3091 (4.2)	3250 (4.4)	3364 (4.6)	4209 (5.7)	
Regular physical activity (N (%))						<0.001
None	71,361 (24.3)	16,702 (22.7)	18,048 (24.6)	18,324 (24.9)	18,287 (24.9)	
1–4 days/week	132,267 (45.0)	32,191 (43.8)	32,435 (44.1)	33,222 (45.2)	34,419 (46.8)	
≥5 days/week	90,340 (30.7)	24,599 (33.5)	23,009 (31.3)	21,946 (29.9)	20,786 (28.3)	
Laboratory findings (Mean ± SD)						
AST (U/L)	26.2 ± 15.7	25.2 ± 16.2	25.6 ± 14.0	26.2 ± 14.0	27.9 ± 18.2	<0.001
ALT (U/L)	25.1 ± 18.8	21.6 ± 17.2	23.9 ± 17.8	25.8 ± 18.5	29.3 ± 20.7	<0.001
Total-C (mg/dL)	200.5 ± 36.9	195.4 ± 34.6	199.5 ± 36.3	202.5 ± 37.3	204.5 ± 38.8	<0.001
HDL-C (mg/dL)	54.7 ± 23.3	65.3 ± 29.3	56.6 ± 23.1	51.3 ± 17.9	45.6 ± 15.6	<0.001
LDL-C (mg/dL)	119.1 ± 35.7	115.8 ± 33.0	122.0 ± 34.4	123.3 ± 35.4	115.4 ± 38.9	<0.001
Triglyceride (mg/dL)	136.9 ± 83.5	77.3 ± 32.7	108.3 ± 40.3	141.6 ± 53.0	220.5 ± 105.0	<0.001
FBG (mg/dL)	100.4 ± 23.9	95.9 ± 18.9	98.9 ± 21.6	101.4 ± 24.2	105.2 ± 28.9	<0.001
Comorbidities (N (%))						
Hypertension	86,042 (29.3)	15,649 (21.3)	20,660 (28.1)	23,823 (32.4)	25,910 (35.3)	<0.001
Diabetes mellitus	35,126 (11.9)	5085 (6.9)	7620 (10.4)	9796 (13.3)	12,625 (17.2)	<0.001
Dyslipidemia	44,945 (15.3)	8269 (11.3)	10,737 (14.6)	12,345 (16.8)	13,594 (18.5)	<0.001
Renal disease	37,455 (12.7)	8217 (11.2)	9088 (12.4)	9701 (13.2)	10,449 (14.2)	<0.001
Liver disease	48,458 (16.5)	10,682 (14.5)	11,696 (15.9)	12,551 (17.1)	13,529 (18.4)	<0.001
Charlson comorbidity index (N (%))						<0.001
0	156,292 (53.2)	40,997 (55.8)	38,991 (53.1)	37,950 (51.6)	38,354 (52.2)	
1	118,857 (40.4)	28,080 (38.2)	29,886 (40.6)	30,739 (41.9)	30,152 (41.0)	
2 or more	18,819 (6.4)	4415 (6.0)	4615 (6.3)	4803 (6.5)	4986 (6.8)	

Abbreviations: TG, triglyceride; HDL, high-density lipoprotein; Q, quartile; SD, standard deviation; N, number; AST, aspartate aminotransferase; ALT, alanine aminotransferase; Total-C, total cholesterol; HDL-C, high-density lipoprotein cholesterol; LDL-C, low-density lipoprotein cholesterol; FBG, fasting blood glucose.

**Table 2:** Results of risk of heart failure considering the TG/HDL ratio as a time-dependent covariate.

Groups	N	Events	Person Years	Incidence Rate (per 1000 Person Years)	Unadjusted	Model 1	Model 2
					HR (95% CI)	HR (95% CI)	HR (95% CI)
Total	293,968	27,852	2,791,350	9.978	1.015 (1.013, 1.018)	1.015 (1.012, 1.017)	1.007 (1.002, 1.011)
DM	64,073	7866	603,696	13.030	1.017 (1.014, 1.020)	1.016 (1.013, 1.019)	1.006 (1.002, 1.010)
Non-DM	229,895	19,986	2,187,654	9.136	1.014 (1.012, 1.017)	1.014 (1.011, 1.017)	1.008 (1.003, 1.013)

Abbreviations: TG, triglyceride; HDL, high-density lipoprotein; N, number; HR, hazard ratio; CI, confidence interval; DM, diabetes mellitus. The estimated HR (95% CI) was calculated using time-dependent Cox regression model. Model 1 was adjusted for age and sex. Model 2 was adjusted for age, sex, body mass index, household income, smoking status, alcohol consumption, regular physical activity, hypertension, diabetes mellitus, dyslipidemia, renal disease, liver disease, and Charlson comorbidity index.

**Table 3:** Risk of heart failure based on the average TG/HDL ratio quartile during the follow-up period.

Average TG/HDL Ratio	N	Events	Person Years	Incidence Rate (per 1000 Person Years)	Unadjusted	Model 1	Model 2
					HR (95% CI)	HR (95% CI)	HR (95% CI)
Total							
Q1 (<1.585)	73,492	5733	700,765	8.181	ref	ref	ref
Q2 (1.585–2.305)	73,492	6921	698,846	9.903	1.212 (1.170, 1.255)	1.176 (1.135, 1.218)	1.093 (1.055, 1.133)
Q3 (2.305–3.403)	73,492	7385	697,261	10.591	1.300 (1.256, 1.346)	1.247 (1.205, 1.291)	1.103 (1.065, 1.143)
Q4 (≥3.403)	73,492	7813	694,477	11.250	1.388 (1.342, 1.436)	1.333 (1.288, 1.380)	1.114 (1.075, 1.155)
*p*-value for trend					<0.001	<0.001	<0.001
DM							
Q1 (<1.991)	16,018	1868	151,024	12.369	ref	ref	ref
Q2 (1.991–2.856)	16,018	1983	151,107	13.123	1.061 (0.996, 1.130)	1.061 (0.996, 1.130)	1.053 (0.993, 1.112)
Q3 (2.856–4.138)	16,018	1989	150,797	13.190	1.069 (1.004, 1.139)	1.075 (1.009, 1.145)	1.064 (1.013, 1.114)
Q4 (≥4.138)	16,019	2026	150,768	13.438	1.089 (1.023, 1.160)	1.108 (1.040, 1.181)	1.102 (1.043, 1.162)
*p*-value for trend					0.009	0.002	0.003
Non-DM							
Q1 (<1.504)	57,474	4170	548,939	7.596	ref	ref	ref
Q2 (1.172–2.172)	57,473	4960	547,917	9.052	1.191 (1.143, 1.242)	1.156 (1.109, 1.204)	1.084 (1.040, 1.130)
Q3 (2.172–3.183)	57,474	5286	546,330	9.675	1.279 (1.228, 1.332)	1.225 (1.176, 1.276)	1.111 (1.066, 1.158)
Q4 (≥3.183)	57,474	5570	544,469	10.230	1.358 (1.305, 1.414)	1.299 (1.248, 1.353)	1.134 (1.087, 1.182)
*p*-value for trend					<0.001	<0.001	<0.001

Abbreviations: TG, triglyceride; HDL, high-density lipoprotein; N, number; HR, hazard ratio; CI, confidence interval; Q, quartile; DM, diabetes mellitus. The estimated HR (95% CI) was derived from the conventional Cox regression model. Model 1 was adjusted for age and sex. Model 2 was adjusted for age, sex, body mass index, household income, smoking status, alcohol consumption, regular physical activity, hypertension, diabetes mellitus, dyslipidemia, renal disease, liver disease, and Charlson comorbidity index.

## Data Availability

The data used in this study are available in the National Health Insurance Service—National Health Screening Cohort (NHIS-HEALS) database; however, restrictions apply to the public availability of these data, which were used under license for the current study. Requests for access to the NHIS data can be made through the National Health Insurance Sharing Service homepage (http://nhiss.nhis.or.kr/bd/ab/bdaba021eng.do (accessed on 9 March 2024)). For access to the database, a completed application form, research proposal, and application for approval from the institutional review board should be submitted to the inquiry committee of research support at the NHIS for review.

## Supplementary Materials

The following supporting information can be downloaded at https://www.mdpi.com/article/10.3390/jcm14030950/s1, Supplementary Methods; Table S1: The number of health screenings conducted during the follow-up period. Table S2: Temporal changes of variables throughout the follow-up period. Table S3: Results of risk of heart failure considering the TG/HDL ratio as a time-dependent covariate. Table S4: Risk of heart failure based on the average TG/HDL ratio quartile during the follow-up period.
