# Supplementary material for: Association Between Triglyceride/High-Density Lipoprotein Ratio and Incidence Risk of Heart Failure: A Population-Based Cohort Study
Source: J Clin Med. 2025 Feb 1;14(3):950. doi: 10.3390/jcm14030950 (PMC11818675; doi:10.3390/jcm14030950)
Supplement: Supplementary file 1 [file jcm-14-00950-s001.zip › jcm-3373907-supplementary.pdf]

Supplementary Methods

Definition of covariates

Smoking history (never, former, and current)

Definition and measurement of variables like usual smoking habits were obtained by questionnaire in the health examination program. Smoking status was used to categorize participants into three groups: none, former smoker, and current smoker. Current smoker was classified according to the WHO definition as a person who has smoked more than five packs (100 cigarettes) in a lifetime and smoked daily or occasionally for the last 28 days. Former smoker was defined as a person who had smoked more than 100 cigarettes in a lifetime and had not smoked in the last 28 days.

Alcohol consumption (none, moderate, and heavy)

Definition and measurement of variables like usual alcohol consumption were obtained by questionnaire in the health examination program. Alcohol consumption was categorized into three groups: none, moderate drinker, and heavy drinker. Differentiation between moderate and heavy drinker was based on whether a patient usually takes more than 14 drinks/7 drinks per week for men/women. The drinks were calculated by multiplying the average drinking frequency per week by the number of drinks per occasion.

Physical activity (low, moderate)

Physical activity was assessed using the Korean version of the International Physical Activity Questionnaire-short form. We created composite physical activity based on Metabolic Equivalent Task (MET)-minutes/week (walking: 3.3 METs; moderate physical activity: 4.0 METs; vigorous physical activity: 8.0 METs), which was categorized as follows based on total physical activity metabolic equivalents: low (< 600 METs), moderate (600–2,999 METs), and vigorous (≥ 3,000 METs).

Comorbidities

Hypertension

Hypertension was defined as using at least one claim of ICD-10 code(I10-15) with the prescription of an anti-hypertensive agent, claims of ICD-10 code(I10-15) more than two times, a systolic blood pressure of ≥140 mmHg and a diastolic blood pressure of ≥90 mmHg

or positive checking in self-report questionnaire on hypertension in the health examination program.

Diabetes mellitus

Diabetes mellitus was as defined using at least one claim of ICD-10 code(E11-14) with the prescription of an anti-diabetic agent, claims of ICD-10 code(E11-14) more than two times, fasting serum glucose concentration of ≥7.0 mmol/L or positive checking in self-report questionnaire on diabetes mellitus in the health examination program.

Dyslipidemia

Dyslipidemia was defined as using at least one claim of ICD-10 code(E78) with the prescription of an anti-dyslipidemic agent, claims of ICD-10 code(E78) more than two times or total cholesterol level of ≥240 mg/dL.

Renal disease

Renal disease was defined as using claims of ICD–10 codes (N17-19, I12-13, E08.2, E10.2, E11.2, E13.2) more than two times or estimated glomerular filtration rate (eGFR) of <60 mL/min/1.73m^2.

Liver disease

Liver disease was defined as using claims of ICD-10 codes (B18, K70, K71, K72, K73, K74, K76.1) with more than two times of admission or outpatient department.

Charlson comorbidity index (0, 1, or ≥2).

The Charlson comorbidity index score was calculated for each subject based on diseases diagnosed before index date and divided into three groups (0, 1, and ≥2 scores).

Table S1. The number of health screenings conducted during the follow-up period.

Number of measurements	Total (n=293,968)	DM (n=64,073)	Non-DM (n=229,895)
3	42,503 (14.46%)	10,851 (16.94%)	31,652 (13.77%)
4	76,550 (26.04%)	17,534 (27.37%)	59,016 (25.67%)
5	78,158 (26.59%)	16,542 (25.82%)	61,616 (26.80%)
6	38,325 (13.04%)	7,890 (12.31%)	30,435 (13.24%)
7	21,337 (7.26%)	4,342 (6.78%)	16,995 (7.39%)
8	23,883 (8.12%)	4,452 (6.95%)	19,431 (8.45%)
9	12,869 (4.37%)	2,387 (3.71%)	10,482 (4.56%)
10	343 (0.12%)	75 (0.12%)	268 (0.12%)

Abbreviations: N, number; DM, diabetes mellitus.

Table S2. Temporal Changes of variables throughout the follow-up period.

Variables	2009, (N=214,281)	2010, (N=219,219)	2011, (N=218,889)	2012, (N=211,229)	2013, (N=200,230)	2014, (N=204,434)	2015, (N=216,149)	2016, (N=199,799)	2017, (N=207,233)	2018, (N=199,597)	2019, (N=208,172)
Age, years											
< 65	167,872 (78.3)	173,877 (79.3)	164,953 (75.4)	161,489 (76.5)	142,969 (71.4)	147,631 (72.2)	145,590 (67.4)	126,060 (63.1)	120,181 (58.0)	112,294 (56.3)	105,713 (50.8)
≥ 65	46,409 (21.7)	45,342 (20.7)	53,936 (24.6)	49,740 (23.5)	57,261 (28.6)	56,803 (27.8)	70,559 (32.6)	73,739 (36.9)	87,052 (42.0)	87,303 (43.7)	102,459 (49.2)
Sex, N (%)											
Female	94,738 (44.2)	91,021 (41.5)	96,971 (44.3)	88,145 (41.7)	89,304 (44.6)	86,162 (42.1)	95,148 (44.0)	85,635 (42.9)	92,959 (44.9)	86,352 (43.3)	94,930 (45.6)
Male	119,543 (55.8)	128,198 (58.5)	121,918 (55.7)	123,084 (58.3)	110,926 (55.4)	118,272 (57.9)	121,001 (56.0)	114,164 (57.1)	114,274 (55.1)	113,245 (56.7)	113,242 (54.4)
Body mass index (kg/m2)											
< 25	140,192 (65.4)	143,894 (65.6)	143,365 (65.5)	138,853 (65.7)	131,619 (65.7)	133,713 (65.4)	138,788 (64.2)	125,578 (62.9)	128,158 (61.8)	122,069 (61.2)	127,494 (61.2)
≥ 25	74,089 (34.6)	75,325 (34.4)	75,524 (34.5)	72,376 (34.3)	68,611 (34.3)	70,721 (34.6)	77,361 (35.8)	74,221 (37.1)	79,075 (38.2)	77,528 (38.8)	80,678 (38.8)
Waist circumference (cm)											
Male < 90, female < 85	163,426 (76.3)	168,698 (77.0)	167,089 (76.3)	162,119 (76.8)	151,692 (75.8)	155,021 (75.8)	159,504 (73.8)	145,682 (72.9)	149,367 (72.1)	143,061 (71.7)	146,731 (70.5)
Male ≥ 90, female ≥ 85	50,855 (23.7)	50,521 (23.0)	51,800 (23.7)	49,110 (23.2)	48,538 (24.2)	49,413 (24.2)	56,645 (26.2)	54,117 (27.1)	57,866 (27.9)	56,536 (28.3)	61,441 (29.5)
Household income, N (%)											
Low	144,161 (67.3)	137,480 (62.7)	148,053 (67.6)	130,303 (61.7)	132,974 (66.4)	130,163 (63.7)	143,052 (66.2)	132,528 (66.3)	138,966 (67.1)	134,153 (67.2)	140,175 (67.3)
High	70,120 (32.7)	81,739 (37.3)	70,836 (32.4)	80,926 (38.3)	67,256 (33.6)	74,271 (36.3)	73,097 (33.8)	67,271 (33.7)	68,267 (32.9)	65,444 (32.8)	67,997 (32.7)
Smoking status, N (%)											
Never	135,217 (63.1)	133,805 (61.0)	136,742 (62.5)	128,487 (60.8)	125,693 (62.8)	125,494 (61.4)	136,207 (63.0)	123,939 (62.0)	132,212 (63.8)	124279 (62.3)	128964 (62.0)
Former	39,588 (18.5)	45,053 (20.6)	45,105 (20.6)	46,557 (22.0)	42,836 (21.4)	46,950 (23.0)	51,999 (24.1)	49,036 (24.5)	50,231 (24.2)	48275 (24.2)	50962 (24.5)
Current	39,476 (18.4)	40,361 (18.4)	37,042 (16.9)	36,185 (17.2)	31,701 (15.8)	31,990 (15.6)	27,943 (12.9)	26,824 (13.5)	24,790 (12.0)	26930 (13.5)	28081 (13.5)
Alcohol consumption (days/week), N (%)											
None	126,066 (58.8)	126,771 (57.8)	131,606 (60.1)	123,923 (58.7)	122,946 (61.4)	122,277 (59.8)	133,240 (61.6)	123,438 (61.8)	132,348 (63.9)	128069 (64.2)	133749 (64.3)
1-2 times	57,930 (27.0)	61,311 (28.0)	56,583 (25.8)	57,921 (27.4)	50,448 (25.2)	54,700 (26.8)	54,590 (25.3)	50,559 (25.3)	49,692 (24.0)	47477 (23.8)	49090 (23.6)
3-4 times	19,721 (9.3)	20,899 (9.5)	20,041 (9.2)	19,731 (9.3)	17,579 (8.8)	18,332 (9.0)	18,821 (8.7)	17,161 (8.6)	16,462 (7.9)	15360 (7.7)	16433 (7.9)
≥ 5 times	10,564 (4.9)	10,238 (4.7)	10,659 (4.9)	9,654 (4.6)	9,257 (4.6)	9,125 (4.4)	9,498 (4.4)	8,641 (4.3)	8,731 (4.2)	8578 (4.3)	8736 (4.2)
Regular physical activity (days/week) , N (%)											
None	55,403 (25.9)	52,208 (23.8)	53,261 (24.4)	47,736 (22.6)	46,095 (23.0)	42,353 (20.7)	44,617 (20.6)	39,040 (19.5)	39,083 (18.9)	36306 (18.2)	37649 (18.1)
1-4 days	94,882 (44.2)	99,827 (45.6)	95,711 (43.7)	96,328 (45.6)	88,190 (44.1)	93,408 (45.7)	95,668 (44.3)	88,226 (44.2)	89,324 (43.1)	86576 (43.4)	89859 (43.2)
≥ 5 days	63,996 (29.9)	67,184 (30.6)	69,917 (31.9)	67,165 (31.8)	65,945 (32.9)	68,673 (33.6)	75,864 (35.1)	72,533 (36.3)	78,826 (38.0)	76602 (38.4)	80499 (38.7)
Laboratory findings											
AST (U/L)	26.5 ± 15.7	26.4 ± 16.8	26.4 ± 14.8	26.7 ± 17.6	26.6 ± 15.9	26.7 ± 16.2	27.1 ± 15.7	27.0 ± 15.9	27.4 ± 17.0	27.6 ± 18.5	27.8 ± 27.6
ALT (U/L)	25.3 ± 19.2	25.2 ± 18.4	24.5 ± 17.9	24.9 ± 19.3	24.6 ± 18.2	24.9 ± 18.5	25.1 ± 18.5	25.1 ± 18.6	25.0 ± 19.8	25.1 ± 20.2	24.8 ± 20.7
Total-C (mg/dL)	199.9 ± 37.4	199.3 ± 37.0	198.0 ± 37.3	197.7 ± 37.2	197.1 ± 37.7	195.4 ± 37.9	194.7 ± 38.8	194.5 ± 39.4	192.4 ± 39.8	190.8 ± 40.3	187.8 ± 40.7
HDL-C (mg/dL)	55.1 ± 29.0	54.0 ± 17.4	54.1 ± 15.6	54.1 ± 17.2	53.7 ± 15.3	53.6 ± 14.4	54.1 ± 14.5	54.4 ± 15.3	54.8 ± 14.2	55.1 ± 17.4	55.2 ± 14.4
LDL-C (mg/dL)	118.1 ± 36.6	118.3 ± 35.1	117.3 ± 34.6	117.3 ± 34.6	116.9 ± 34.9	115.8 ± 35.2	114.9 ± 35.4	114.4 ± 36.1	111.7 ± 36.3	110.2 ± 36.9	107.8 ± 36.9
Triglyceride (mg/dL)	140.4 ± 89.1	136.6 ± 80.4	134.2 ± 78.2	133.7 ± 80.4	133.7 ± 80.1	132.2 ± 79.4	130.7 ± 78.6	130.8 ± 78.4	130.7 ± 78.1	130.1 ± 77.5	124.9 ± 71.3
FBG (mg/dL)	100.8 ± 25.4	101.1 ± 25.1	101.3 ± 24.8	101.7 ± 24.9	102.6 ± 25.4	103.3 ± 25.5	103.9 ± 25.6	104.6 ± 25.7	105.0 ± 25.8	105.5 ± 25.4	105.9 ± 25.3
Comorbidities											
Hypertension, N (%)	81,422 (38.0)	82,924 (37.8)	88,319 (40.3)	85,497 (40.5)	85,650 (42.8)	86,499 (42.3)	95,238 (44.1)	79,274 (39.7)	86,142 (41.6)	85,141 (42.7)	92,755 (44.6)
Diabetes mellitus, N (%)	30,136 (14.1)	30,796 (14.0)	33,180 (15.2)	32,505 (15.4)	33,411 (16.7)	34,748 (17.0)	38,897 (18.0)	36,201 (18.1)	39,659 (19.1)	39,876 (20.0)	43,651 (21.0)
Dyslipidemia, N (%)	58,915 (27.5)	62,831 (28.7)	68,438 (31.3)	70,094 (33.2)	73,659 (36.8)	78,236 (38.3)	91,505 (42.3)	91,421 (45.8)	101,695 (49.1)	93,967 (47.1)	106,467 (51.2)
Renal disease, N (%)	32,061 (15.0)	28,981 (13.2)	26,591 (12.1)	22,743 (10.8)	23,215 (11.6)	21,989 (10.8)	23,664 (10.9)	21,343 (10.7)	23,259 (11.2)	22,914 (11.5)	25,245 (12.1)
Liver disease, N (%)	34,654 (16.2)	36,651 (16.7)	39,370 (18.0)	39,989 (18.9)	41,130 (20.5)	42,479 (20.8)	48,841 (22.6)	48,409 (24.2)	53,458 (25.8)	58,227 (29.2)	67,838 (32.6)
Charlson comorbidity index, N (%)											
0	213,991 (99.9)	218,927 (99.9)	218,650 (99.9)	211,020 (99.9)	200,076 (99.9)	204,232 (99.9)	215,994 (99.9)	199,661 (99.9)	207,080 (99.9)	199,479 (99.9)	208,073 (99.9)
1	246 (0.1)	247 (0.1)	200 (0.1)	170 (0.1)	131 (0.1)	171 (0.1)	132 (0.1)	116 (0.1)	126 (0.1)	107 (0.1)	78 (0.1)
2 or more	44 (0.0)	45 (0.0)	39 (0.0)	39 (0.0)	23 (0.0)	31 (0.0)	23 (0.0)	22 (0.0)	27 (0.1)	11 (0.0)	21 (0.0)

Abbreviations: N, number; AST, aspartate aminotransferase; ALT, alanine aminotransferase; Total-C, total cholesterol; HDL-C, high-density lipoprotein cholesterol; LDL-C, low-density lipoprotein cholesterol; FBS, fasting blood glucose.

Table S3. Results of risk of heart failure considering the TG/HDL ratio as a time-dependent covariate.

Variables	Total N=293,968		DM N=64,073		Non-DM N=229,895	
	Model 1 HR (95% CI)	Model 2 HR (95% CI)	Model 1 HR (95% CI)	Model 2 HR (95% CI)	Model 1 HR (95% CI)	Model 2 HR (95% CI)
TG/HDL ratio	1.015 (1.012, 1.017)	1.007 (1.002, 1.011)	1.016 (1.013, 1.019)	1.006 (1.002, 1.010)	1.014 (1.011, 1.017)	1.008 (1.003, 1.013)
Age, years						
< 65	ref	ref	ref	ref	ref	ref
≥ 65	1.489 (1.453, 1.526)	1.152 (1.123, 1.181)	1.313 (1.251, 1.378)	1.117 (1.062, 1.174)	1.496 (1.454, 1.540)	1.153 (1.119, 1.187)
Sex						
Female	ref	ref	ref	ref	ref	ref
Male	1.172 (1.144, 1.200)	1.288 (1.247, 1.329)	1.081 (1.032, 1.132)	1.239 (1.167, 1.315)	1.171 (1.139, 1.204)	1.303 (1.255, 1.353)
Body mass index (kg/m2)						
< 25		ref		ref		ref
≥ 25		1.068 (1.042, 1.094)		1.053 (1.007, 1.102)		1.070 (1.040, 1.102)
Household income						
Low		ref		ref		ref
High		1.026 (1.001, 1.052)		0.991 (0.945, 1.038)		1.039 (1.010, 1.07)
Smoking status						
Never		ref		ref		ref
Former		0.984 (0.950, 1.020)		0.954 (0.895, 1.018)		1.000 (0.959, 1.043)
Current		1.085 (1.040, 1.132)		1.055 (0.978, 1.137)		1.104 (1.048, 1.162)
Alcohol consumption (days/week)						
None		ref		ref		ref
1-2 times		0.776 (0.750, 0.803)		0.732 (0.687, 0.781)		0.793 (0.762, 0.825)
3-4 times		0.839 (0.799, 0.882)		0.727 (0.662, 0.798)		0.883 (0.833, 0.936)
≥ 5 times		0.978 (0.924, 1.034)		0.938 (0.848, 1.037)		0.984 (0.919, 1.053)
Regular physical activity (days/week)						
None		ref		ref		ref
1-4 days		0.762 (0.740, 0.785)		0.738 (0.698, 0.781)		0.775 (0.747, 0.803)
≥ 5 days		0.718 (0.697, 0.741)		0.684 (0.646, 0.724)		0.735 (0.708, 0.762)
Hypertension		1.354 (1.300, 1.411)		1.423 (1.321, 1.533)		1.315 (1.253, 1.381)
Diabetes mellitus		0.858 (0.834, 0.882)				
Dyslipidemia		1.359 (1.323, 1.395)		1.046 (0.992, 1.104)		1.457 (1.414, 1.500)
Renal disease		1.207 (1.164, 1.251)		1.320 (1.249, 1.396)		1.181 (1.127, 1.238)
Liver disease		1.044 (1.014, 1.075)		1.076 (1.024, 1.130)		1.030 (0.994, 1.067)
Charlson comorbidity index		2.027 (1.999, 2.055)		1.658 (1.606, 1.712)		2.127 (2.095, 2.160)

Abbreviations: TG, triglyceride; HDL, high-density lipoprotein; N, number; HR, hazard ratio; CI, confidence interval; DM, diabetes mellitus. The estimated HR (95% CI) was derived from the time-dependent Cox regression model, with the TG/HDL ratio being considered as a time-dependent covariate, and the last time point of follow-up information was used for other adjustment variables.

Table S4. Risk of heart failure based on the average TG/HDL ratio quartile during the follow-up period.

Variables	Total N=293,968 Model 1 HR (95% CI)	Total N=293,968 Model 2 HR (95% CI)	DM N=64,073 Model 1 HR (95% CI)	DM N=64,073 Model 2 HR (95% CI)	Non-DM N=229,895 Model 1 HR (95% CI)	Non-DM N=229,895 Model 2 HR (95% CI)
Average TG/HDL ratio						
Q1	ref	ref	ref	ref	ref	ref
Q2	1.176 (1.135, 1.218)	1.093 (1.055, 1.133)	1.061 (0.996, 1.130)	1.053 (0.993, 1.112)	1.156 (1.109, 1.204)	1.084 (1.040, 1.130)
Q3	1.247 (1.205, 1.291)	1.103 (1.065, 1.143)	1.075 (1.009, 1.145)	1.064 (1.013, 1.114)	1.225 (1.176, 1.276)	1.111 (1.066, 1.158)
Q4	1.333 (1.288, 1.380)	1.114 (1.075, 1.155)	1.108 (1.040, 1.181)	1.102 (1.043, 1.162)	1.299 (1.248, 1.353)	1.134 (1.087, 1.182)
Age, years						
< 65	ref	ref	ref	ref	ref	ref
≥ 65	1.478 (1.442, 1.514)	1.148 (1.120, 1.178)	1.316 (1.255, 1.381)	1.117 (1.062, 1.174)	1.485 (1.444, 1.528)	1.149 (1.115, 1.183)
Sex						
Female	ref	ref	ref	ref	ref	ref
Male	1.144 (1.117, 1.172)	1.280 (1.24, 1.322)	1.075 (1.027, 1.126)	1.238 (1.167, 1.315)	1.146 (1.114, 1.179)	1.293 (1.245, 1.342)
Body mass index (kg/m2)						
< 25		ref		ref		ref
≥ 25		1.058 (1.032, 1.084)		1.052 (1.005, 1.100)		1.057 (1.027, 1.089)
Household income						
Low		ref		ref		ref
High		1.026 (1.001, 1.052)		0.991 (0.945, 1.038)		1.040 (1.010, 1.070)
Smoking status						
Never		ref		ref		ref
Former		0.982 (0.948, 1.017)		0.954 (0.895, 1.017)		0.996 (0.955, 1.039)
Current		1.077 (1.032, 1.124)		1.053 (0.977, 1.136)		1.093 (1.038, 1.151)
Alcohol consumption (days/week)						
None		ref		ref		ref
1-2 times		0.777 (0.752, 0.804)		0.733 (0.687, 0.781)		0.794 (0.763, 0.826)
3-4 times		0.842 (0.801, 0.884)		0.727 (0.662, 0.798)		0.886 (0.836, 0.939)
≥ 5 times		0.982 (0.929, 1.039)		0.938 (0.849, 1.038)		0.989 (0.924, 1.058)
Regular physical activity (days/week)						
None		ref		ref		ref
1-4 days		0.764 (0.741, 0.787)		0.738 (0.698, 0.781)		0.776 (0.749, 0.805)
≥ 5 days		0.721 (0.699, 0.744)		0.684 (0.646, 0.724)		0.738 (0.712, 0.766)
Hypertension		1.352 (1.298, 1.408)		1.422 (1.320, 1.533)		1.312 (1.250, 1.378)
Diabetes mellitus		0.852 (0.828, 0.876)				
Dyslipidemia		1.351 (1.316, 1.387)		1.046 (0.991, 1.104)		1.447 (1.405, 1.490)
Renal disease		1.204 (1.162, 1.248)		1.319 (1.248, 1.395)		1.177 (1.123, 1.234)
Liver disease		1.045 (1.015, 1.075)		1.076 (1.024, 1.130)		1.031 (0.995, 1.069)
Charlson comorbidity index		2.027 (2.000, 2.055)		1.658 (1.606, 1.712)		2.128 (2.096, 2.161)

Abbreviations: TG, triglyceride; HDL, high-density lipoprotein; N, number; HR, hazard ratio; CI, confidence interval; Q, quartile; DM, diabetes mellitus. The estimated HR (95% CI) was derived from the conventional Cox regression model, wherein the TG/HDL ratio was considered as the quartiles of its average during the follow-up period, and the last time point of follow-up information was used for other adjustment variables.
